# Association of treated neovascular AMD with disability and mood disorders: A longitudinal nationwide study

**DOI:** 10.1111/aos.70130

**Published:** 2026-04-18

**Authors:** Cécile Delcourt, Emilie Hucteau, Sarra Gattoussi, Karine Pérès, Marie‐Noelle Delyfer, Catherine Helmer, Jean‐François Korobelnik

**Affiliations:** ^1^ Univ. Bordeaux, INSERM, BPH, U219 Bordeaux France; ^2^ Service d'Ophtalmologie, CHU de Bordeaux Bordeaux France

**Keywords:** age‐related macular degeneration, burden, disability, falls, mental health

## Abstract

**Purpose:**

To describe the associations of treated neovascular AMD with the risk of disability, falls, fractures and mood disorders in a large sample representative of the French population.

**Methods:**

Analyses were based on 77 582 individuals belonging to the ‘*Echantillon Généraliste des Bénéficiaires*’, a 1/97 random sample of the French population, aged 65 years or more on 1 April 2009 and followed until 31 December 2019. Treated neovascular AMD (nAMD) was identified through the reimbursement of anti‐VEGF drugs, intravitreal injections and after exclusion of other pathologies requiring the use of intravitreal anti‐VEGFs. Activities of daily living (ADL) disability was identified using a previously developed algorithm, while falls, fractures, head trauma and mood disorders were identified using relevant ICD codes and medications. Associations of nAMD with outcomes were assessed using Cox models with nAMD as a time‐dependent variable and adjustment for confounders.

**Results:**

During the study period, 1662 cases of incident nAMD were identified. After adjustment for age, sex, Charlson comorbidity index and number of drugs, nAMD was significantly associated with the occurrence of ADL disability (Hazard ratio (HR): 1.19, 95% confidence interval (CI): 1.08–1.30; *p* = 0.0002) and of mood disorders (HR: 1.19, 95%CI: 1.07–1.32; *p* = 0.002). Associations with falls, fractures and head trauma were not statistically significant.

**Conclusion:**

Treated neovascular AMD remains associated with a 19% increased risk of disability and mood disorders, while no association was detected with falls, fractures and head trauma. These results are consistent with a major improvement in functional outcomes, with respect to the pre‐anti‐VEGF era.

## 1 INTRODUCTION

With the rise in life expectancy and the aging of the global population, maintaining independency in daily living activities in older adults is a major challenge for our societies. Vision being involved in most activities of daily living (shopping, using transportation, dressing, using the telephone, managing medications…), visual impairment is associated with an increased risk of disability, in particular in individuals with visual acuity below 20/40 (Daien et al., 2011; Nael et al., 2017; Peres et al., 2017). Visual impairment is also associated with falls and fractures (Lamoureux et al., 2008; Patino et al., 2010; Yip et al., 2014), as well as mood disorders, especially depression (Chou, 2008; Cosh et al., 2018, 2019; Evans et al., 2007; Zhang et al., 2013), which may further increase the risk of disability.

Advanced AMD (atrophic and/or neovascular) is naturally associated with major visual impairment and a leading cause of blindness in industrialized countries (GBD 2019 Blindness, 2021). However, since their first authorization in 2007, treatments with intravitreal anti‐vascular endothelial growth factor (VEGF) agents have profoundly changed the visual prognostic of neovascular AMD (nAMD). Indeed, they result in a mean gain of about 7 ETDRS letters at 2 years, compared with a mean loss of 15 ETDRS letters in the placebo arm in the pivotal trials (Rosenfeld et al., 2006; Schmidt‐Erfurth et al., 2014). Real‐world studies confirmed the initial gain in vision with anti‐VEGF therapy, with about half of the patients attaining a best‐corrected visual acuity (BCVA) of at least 20/40 at 2 years (Barthelmes et al., 2018; Fu et al., 2021). However, visual impairment cannot always be avoided over the long run, because of insufficient treatment efficacy and/or the occurrence of retinal atrophy in one or both eyes, although half of the patients retained a BCVA greater than 20/200 after 8 years in a retrospective study (Fu et al., 2021).

A few studies from the pre‐anti‐VEGF era have shown a major increased risk of disability in patients with advanced AMD and in particular nAMD. In an international case–control study performed in 2005, assistance in activities of daily living was 4 times more frequent in patients with bilateral nAMD than in controls (Soubrane et al., 2007). The rate of falls was also twice as high in cases, and scores of anxiety and depression were 30% and 42% higher (Soubrane et al., 2007). In the Blue Mountains Eye Study, advanced AMD at baseline (2002–2004) was associated with a 13‐fold increased risk of incident disability at 5 years (2007–2009). However, there is a lack of recent data in nAMD treated with anti‐VEGF agents.

The objective of the present study was to describe the associations of treated neovascular AMD with the risk of disability, mood disorders, falls and fractures in a large sample, representative of the French population.

## 2 METHODS

### 2.1 Study population and data collection

The ‘*Echantillon Généraliste des Bénéficiaires*’ (EGB) is an anonymous permanent sample, representative in age and sex of the total French population covered by the Health Insurance schemes (Tuppin et al., 2010). It is a 1/97 survey based on health insurance beneficiaries with data historization since 2005. It includes about 810 000 beneficiaries and contains information on their sociodemographic and medical characteristics and the benefits they have received. A linkage with the PMSI MCO (Medicine, Surgery, Obstetrics) and HAD (at home hospitalization) also makes it possible to track their hospitalizations. For this study, we used the EGB from 2009 to 2019. By agreement of the French Data Protection Supervisory Authority (CNIL), neither ethics committee approval nor informed consent was required for this observational study based on anonymised French medico‐administrative databases.

The study population consisted of all individuals aged 65 years or older affiliated to the General Health Insurance Scheme (covering about 85% of the French population) on 1 April 2009. Persons with nAMD identified during the first quarter of 2009 were excluded as well as persons with no follow‐up.

For the main analysis, persons with prevalent activities of daily living (ADL) disability as of the first quarter of 2009 were excluded. The same criteria were used to select the study populations for the secondary analyses, replacing ADL disability by each of the four other outcomes: falls, fractures, head traumas and mood disorders.

### 2.2 Definition of neovascular AMD

To identify persons with nAMD, an algorithm was developed on the EGB. It was identified through the reimbursement of anti‐VEGF drugs, intravitreal injections and after exclusion of other pathologies requiring the use of intravitreal anti‐VEGFs (from other causes than nAMD). The algorithm codes used to define each variable are detailed in Tables S1 and S2. Among the 77 582 individuals aged 65 or more and affiliated to the General Scheme of the Health Insurance system on 1 April 2009, 2518 had at least 2 reimbursements at different dates of anti‐VEGF agents within a 90 days period, combined with at least 2 reimbursements in the same year for intravitreal injections, during the follow‐up period (Figure S1). Among them, 244 individuals with ICD‐10 codes for macular oedema, retinal vein occlusion, high myopia (based on reimbursement of eyeglasses with lenses for high myopia), choroidal neovascularization (from other causes than nAMD) or treated with Ozurdex (which is indicated for diabetic macular oedema and retinal vein occlusion) were classified as no nAMD. In addition, we included in the no nAMD group those with diabetic eye complications (diabetic macular oedema, laser for diabetic retinopathy). Firstly, 417 individuals were identified as affected by diabetes, according to the algorithm developed by the ‘*Caisse Nationale d'Assurance Maladie (CNAM)*’ (DEPP, 2022). Among them, 126 had codes for diabetic macular oedema or laser for diabetic retinopathy and were included in the no nAMD group. The other 291 individuals with diabetes but without diabetic eye complications were considered as having nAMD. Overall, excluding 133 prevalent nAMD during 2009 first quarter, 2015 individuals were classified as having nAMD during the follow‐up period.

Exposure to nAMD was assessed during the follow‐up from 2009 to 2019. This variable was time‐dependent: (i) nAMD = 0 until the first date of identification of an anti‐VEGF drug reimbursement or until the end of follow‐up if no anti‐VEGF reimbursement; (ii) nAMD = 1 from the first date of identification of a reimbursement of anti‐VEGF drugs to the end of follow‐up (ADL disability, death or 31 December 2019).

### 2.3 ADL disability and secondary outcomes

Events of interest were considered between 1 April 2009 and 31 December 2019. For the main analysis, the event of interest was the occurrence of activities of daily living (ADL)‐disability, which was defined using an algorithm developed on the EGB database by our team (Hucteau et al., 2021). It included data on drugs, long‐term diseases, acts and equipment and products, age and sex and was designed to be applied quarter by quarter. Since ADL disability is assessed by this algorithm once a quarter, the reference date corresponded to the first day of the first quarter during which the individual became dependent.

For the secondary analyses, the events of interest were, respectively, falls, fractures, head injuries and mood disorders (suicide attempt or psychotropic drugs). Each event was defined based on specific ICD codes for hospitalization or drug deliveries and the first occurrence of the event was considered (Table S2).

### 2.4 Other variables

Other variables were used to describe the selected population or to adjust the survival analysis: sex, age at inclusion (1 January 2009), number of drugs at inclusion, calculated as the number of different ATC codes delivered the year before the inclusion and the Charlson comorbidity index at inclusion (Bannay et al., 2016).

### 2.5 Statistical analysis

Baseline characteristics of the study population were described with a comparison between incident nAMD and never nAMD individuals.

To assess the associations of nAMD with ADL disability, proportional hazard Cox models were performed. End of the follow‐up corresponded to the first between the date of occurrence of the outcome, the end of affiliation to the general scheme of Health Insurance, the date of death or the end of the follow‐up.

In the Cox models, nAMD was considered as a time‐dependent variable as nAMD status could change during the follow‐up. Different levels of adjustment were used: M1: age, sex; and M2: M2 + Charlson comorbidity index and number of medications. Results are presented as hazard ratio (HR) and 95% confidence interval (95%CI).

Secondary analyses were performed to assess the associations of nAMD with the risk of falls, fractures, head trauma and mood disorders, using the same Cox modelling. For each outcome, prevalent cases at baseline (i.e. during the first quarter of 2009) were excluded.

As diabetic macular oedema is the most frequent alternative indication of anti‐VEGF agents, we performed a sensitivity analysis excluding all diabetic patients (not only those with diabetic eye complications). In addition, we performed another sensitivity analysis excluding ADL‐disabled individuals and those with nAMD in 2009, in order to exclude more strictly prevalent cases.

The statistical analysis was performed with SAS Enterprise Guide^®^ software (SAS Institute, version 9.4, North Carolina, USA).

## 3 RESULTS

**FIGURE 1:**
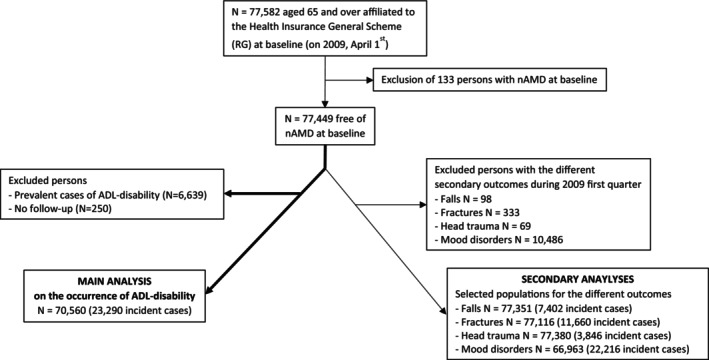
Flowchart for selection of individuals in analyses.

Among the 77 582 individuals aged 65 and over affiliated to the General Health Insurance, 77 449 individuals were included in the study population, after exclusion of 133 prevalent cases of nAMD at baseline (2009 first quarter) (Figure 1).

### 3.1 Main analysis: nAMD and ADL disability

The main analysis was performed on 70 560, after exclusion of the 6639 individuals with prevalent ADL disability at baseline (2009 first quarter) and 250 individuals without follow‐up. Among them, 1662 persons had an incident nAMD during the follow‐up, with a mean age of nAMD detected at 82.0 years (Table 1). The mean age at baseline of the persons with incident nAMD was 76.3 years vs. 75.1 years among never nAMD persons. There were more women among nAMD persons (65.2% vs. 57.3%). About 70% of the sample was at low risk of dying in 2009 (Charlson index of 0) and persons with incident nAMD consumed slightly more drugs at baseline than persons without.

**TABLE 1:** Baseline sociodemographic and medical characteristics of included persons according to their nAMD status during the follow‐up, EGB, main analysis (2009–2019).

Characteristics	Incident nAMD *N* = 1662 *n* (%)	Non‐incident nAMD *N* = 68 898 *n* (%)
Age (years)		
Mean (SD)	76.3 (6.2)	75.1 (7.4)
Median (Q1‐Q3)	76.0 (72.0–81.0)	74.0 (69.0–80.0)
Min‐max	65.0–101.0	65.0–105.0
Age at the beginning of nAMD	82.0 (6.2)	–
Sex		
Men	579 (34.8)	29 399 (42.7)
Women	1083 (65.2)	39 499 (57.3)
Charlson comorbidity index		
Mean (SD)	0.5 (0.8)	0.5 (0.9)
0	1189 (71.5)	49 944 (72.5)
[1–2]	414 (24.9)	16 327 (23.7)
[3–4]	57 (3.4)	2446 (3.6)
≥5	2 (0.1)	181 (0.3)
Number of drugs		
Mean (SD)	13.4 (8.4)	12.1 (9.1)
≤5	278 (16.7)	17 807 (25.9)
[6–11]	480 (28.9)	17 684 (25.7)
[12–18]	499 (30.0)	18 141 (26.3)
>18	405 (24.4)	15 266 (22.2)

Abbreviations: EGB ‘*Echantillon Généraliste des Bénéficiaires*’, a 1/97 random sample of the French population; SD, standard deviation.

During the follow‐up, there were 23 290 incident cases of ADL disability. nAMD was significantly associated with the occurrence of ADL disability in the two models: HR: 1.41 (95%CI: 1.29–1.54) in M1 with adjustment for age and sex and HR: 1.19 (95%CI: 1.08–1.30) in M2 with adjustment for age, sex, Charlson comorbidity index and number of drugs (Table 2).

**TABLE 2:** Associations of nAMD with risk of ADL disability and secondary outcomes in Cox modelling, EGB (2009–2019).

	I/T^a^	HR	95%CI	*p*
*Main analysis*				
ADL disability	23 290/70 560			
M1		1.41	1.29–1.54	***<0.0001***
M2		1.19	1.08–1.30	***0.0002***
*Secondary outcomes*				
Mood disorders	22 216/66 963			
M1		1.34	1.20–1.49	***<0.0001***
M2		1.19	1.07–1.32	***0.002***
Falls	7402/77 351			
M1		1.21	1.04–1.39	***0.01***
M2		1.10	0.95–1.27	*0.19*
Fractures	11 660/77 116			
M1		1.20	1.06–1.36	***0.005***
M2		1.12	0.98–1.27	*0.09*
Head trauma	3846/77 380			
M1		1.31	1.08–1.60	**0.01**.
M2		1.19	0.98–1.45	*0.08*

*Note:* Bold and italic *p* values indicate statistical significance (*p* < 0.05).

Abbreviations: CI, confidence interval; EGB: ‘*Echantillon Généraliste des Bénéficiaires*’, a 1/97 random sample of the French population; M1, adjusted for age and sex; M2, adjusted for age, sex, Charlson comorbidity index and number of drugs.

^a^ Incident ADL disability/total.

### 3.2 nAMD and secondary outcomes

Among the 77 449 individuals in the study population, 77 351 were selected for the fall analysis, 77 116 for fractures, 77 380 for head trauma and 66 963 for mood disorders (Figure 1). During follow‐up, there were 22 216 incident mood disorders, 7402 incident falls, 11 660 incident fractures and 3846 incident head trauma.

In the four analyses (Table 2), nAMD was significantly associated with the occurrence of the outcome in models adjusted for age and sex: mood disorders HR: 1.34 (95%CI: 1.20–1.49), falls HR: 1.21 (95%CI: 1.04–1.39); fractures HR: 1.20 (95%CI: 1.06–1.36); head trauma HR: 1.31 (95%CI: 1.08–1.60). However, when also adjusted for Charlson comorbidity index and number of drugs, the association was only significant for the occurrence of mood disorders with a risk about 20% higher among persons with incident nAMD (HR: 1.19, 95%CI: 1.07–1.32; *p* = 0.002).

### 3.3 Sensitivity analyses

When persons with diabetes were excluded or starting the follow‐up in 2010, the associations found in the main and secondary analyses remained the same with hazard ratios obtained which were very similar (data not shown).

## 4 DISCUSSION

In this large population sample of 77 449 older individuals (75 years on average), we analysed the risk of diverse adverse outcomes in treated nAMD individuals compared to their counterparts. Average age at first treatment for nAMD was 82.0 years, and 65.2% of treated nAMD individuals were women (versus 57.3% in individuals without treated nAMD). The main analysis with full adjustment confirmed the excess risk of developing ADL disability in individuals treated for nAMD during follow‐up (Hazard ratio (HR): 1.19, 95% confidence interval (CI): 1.08–1.30, *p* = 0.0002) and also of mood disorders (HR: 1.19, 95%CI: 1.07–1.32; *p* = 0.002). However, nAMD was not significantly to be associated with risk of falls, fractures and head trauma in the fully‐adjusted model.

Similarly to previous studies, individuals with nAMD had a greater risk of disability than controls. However, the strength of the association appears much lower, with a 20% increased risk in the present study, whereas studies of the pre‐anti‐VEGF era reported 4 to 13‐fold increased risks (Gopinath et al., 2014; Soubrane et al., 2007). This might be due to the fact that anti‐VEGF intravitreal injections are effective in reducing visual impairment in AMD, thus reducing the risk of disability.

In this study, nAMD was also significantly associated with a 20% increased risk of mood disorders, assessed by the occurrence of suicide attempts and/or the use of psychotropic drugs (anti‐depressants, anti‐psychotics and thymoregulators). This is consistent with previous observations in neovascular and advanced AMD (Brody et al., 2001; Soubrane et al., 2007), as well as in visually impaired individuals (Carriere et al., 2013; Chou, 2008; Cosh et al., 2018, 2019; Evans et al., 2007; Owsley et al., 2007; Zhang et al., 2013). The potential mechanisms underlying the association of visual impairment with mood disorders include disruption of melatonin secretion due to reduced light absorption, with consequences on circadian rhythms (including sleep–wake patterns and social rhythms) and depression (Cosh et al., 2018, 2019). Visual impairment may also be associated with reduced social interaction, social isolation and loneliness, which are known to favour depression (Cosh et al., 2018).

By contrast, although nAMD was significantly associated with the risk of falls, fractures and head trauma in the age‐ and sex‐adjusted analyses, these associations were attenuated and not statistically significantly in the fully‐adjusted model (HR = 1.10, 1.12 and 1.19, respectively). These observations are consistent with observations in larger databases, where the risk was moderately increased: In a large medico‐administrative database from England, with identification of fall and fractures derived from general practice, hospital and mortality records, diagnosis of AMD was associated with 1.25‐fold and 1.18‐fold increased risks for falls and fractures, respectively (Tsang et al., 2024). Similarly, in a Korean medico‐administrative database, the risk of fractures was 1.09‐fold higher in individuals with a diagnosis of AMD and slightly higher in those with AMD associated with visual disability (adjusted hazard ratio: 1.17) (Yoon et al., 2024). Thus, our study may lack statistical power to evidence such moderate association. In addition, both of these studies included all types of AMD (including atrophic AMD, for which no treatments are available, and non‐treated nAMD), which may explain somewhat higher estimates.

The major strength of our study is the use of the EGB, a large nationwide sample representative of the French population with exhaustive data on diverse health conditions (based on ICD‐10 codes and algorithms) over a long period which allowed having up to 11 years of follow‐up. With this large database, the analysis could be conducted with high statistical power to relate nAMD to ADL disability and medical data to fit the models, although statistical power was higher for ADL disability and mood disorders (>20 000 incident cases) than for falls, fractures and head trauma (7402, 11 660 and 3846, respectively).

However, diagnoses of ADL disability and nAMD were not directly available in this database, so the study required the use of algorithms for their identification. The algorithm for the detection of ADL disability was developed on a population‐based study and previously applied to the EGB database (Hucteau et al., 2021). In particular, this algorithm does not allow identifying disability of instrumental activities of daily living (such as the use of telephone, transportation, cooking or shopping), in which vision is strongly involved and may be more strongly associated with AMD (Nael et al., 2017). The algorithm for nAMD is similar to those used in previous studies regarding the prevalence and incidence of nAMD in the French population (Creuzot Garcher et al., 2022) or the description and estimation of cost of ophthalmological care for nAMD (Creuzot Garcher et al., 2023; Korobelnik et al., 2021). The main difficulty of these algorithms is to distinguish nAMD from the other indications of anti‐VEGF intravitreal therapy (diabetic macular oedema, retinal vein occlusion, myopic choroidal neovascularization…). As diabetic macular oedema is the most frequent alternative indication, we performed a sensitivity analysis excluding all diabetic patients, which did not alter the results.

Other limitations include the fact that among nAMD, only those who benefited from anti‐VEGF therapy could be detected, thus excluding undiagnosed and untreated cases. In addition, atrophic AMD cannot be detected, as there is currently no approved therapy for this form of AMD in France. Finally, no data are available on visual acuity or whether nAMD is unilateral or bilateral. There were also no data on sociodemographic status or lifestyle, which could not be taken into account in the multivariate models and could lead to residual confounding.

In conclusion, in this large nationwide database, treated neovascular AMD remains associated with a 19% increased risk of disability and mood disorders, while no significant association was detected with falls, fractures and head trauma. These results are consistent with a major improvement in functional outcomes, with respect to the pre‐anti‐VEGF era.

## CONFLICT OF INTEREST STATEMENT

EH, SG, KP, CH: none. CD: Consulting fees from Abbvie and Théa Pharma; MND: Consulting fees from Abbvie, Bayer, Novartis, Roche, Horus Pharma. JFK: Consulting fees from Abbvie, Adverum, Apellis, Bayer, Boehringer Ingelheim, Eyepoint Pharma, Ocular Therapeutix, Roche, SeaBeLife, Thea, Carl Zeiss Meditec.

## Supporting information


**Figure S1.** Algorithm used to identify persons with nAMD.

**Table S1.** Codes used to build the aAMD algorithm.

**Table S2.** Codes used to identify secondary outcomes.
