# Supplementary material for: Association of treated neovascular AMD with disability and mood disorders: A longitudinal nationwide study
Source: Acta Ophthalmol. 2026 Apr 18;104(6):e648–54. doi: 10.1111/aos.70130 (PMC13460392; doi:10.1111/aos.70130)
Supplement: Supplementary file 2 [file AOS-104-e648-s002.docx]

Table S2 – Codes used to identify secondary outcomes

| **Secondary outcomes** | **Type of codes** | **Codes** |
| --- | --- | --- |
| **Falls** | ICD-10 PMSI-MCO | W01,  W02,  W05 to W15,  W17,  W18,  W19 |
| **Fractures** | ICD-10 PMSI-MCO | T02,  T08,  T10,  T12,  T142,  S02,  S12,  S22,  S32,  S42,  S52,  S62,  S72,  S82,  S92 |
| **Head traumas** | ICD-10 PMSI-MCO | S02,  S06 |
| **Mood disorders**  Suicide attempt with hospitalization  Psychotropic drugs  *Antidepressants*  *Antipsychotics*  *Thymoregulators* | ICD-10 PMSI-MCO  ATC and CIP | X60 to X84  N06A  N05A  N05AN,  N03AF01,  N03AG02,  N03AX09,  CIP 3400934876233, CIP 3400935444271 |
