# Supplementary material for: Association of treated neovascular AMD with disability and mood disorders: A longitudinal nationwide study
Source: Acta Ophthalmol. 2026 Apr 18;104(6):e648–54. doi: 10.1111/aos.70130 (PMC13460392; doi:10.1111/aos.70130)
Supplement: Supplementary file 3 [file AOS-104-e648-s003.docx]

Table S1 – Codes used to build the aAMD algorithm

| **Variable** | **Type of Code** | **Code** | **Meaning** |
| --- | --- | --- | --- |
| **Anti-VEGF reimbursement** | ATC | S01LA04 | Ranibizumab (Lucentis) |
|  | ATC | S01LA05 | Aflibercept (Eylea) |
|  | ATC | S01LA03 | Pegaptanib |
|  | UCD | 9876544 | Bevacizumab (Avastin) |
| **Intravitreal injection** | acts | BGLB001 | Intra-vitreal injection of a pharmacological agent |
| **Retinal vein occlusion** | CIM-10 | H340 | Transient retinal artery occlusion |
|  | CIM-10 | H341 | Central retinal artery occlusion |
|  | CIM-10 | H342 | Other retinal artery occlusions |
|  | CIM-10 | H348 | Other retinal vascular occlusions |
|  | CIM-10 | H349 | Retinal vascular occlusion, unspecified |
| **Myopia with high correction lenses** | products and deliveries | codes with lens < - 6 diopters |  |
|  | CIM-10 | H330 | Retinal detachment with retinal break |
|  | CIM-10 | H442 | Degenerative myopia |
|  | CIM-10 | H521 | Myopia |
| **Choroidal neovascularization** | CIM-10 | H318 | Other specified disorders of choroid |
| **Macular edema** | CIM-10 | H358 | Other specified retinal disorders |
| **Dexamethasone intravitreal implant** | CIP | 3400949407118 | Ozurdex |
| **Diabetic macular edema** | CIM-10 | E103 | Type 1 diabetes mellitus with ophtalmic complications |
|  | CIM-10 | E113 | Type 2 diabetes mellitus with ophtalmic complications |
|  | CIM-10 | E123 | Malnutrition-related diabetes mellitus with ophtalmic complications |
|  | CIM-10 | E133 | Other specified diabetes mellitus with ophtalmic complications |
|  | CIM-10 | E143 | Unspecified diabetes mellitus with ophtalmic complications |
|  | CIM-10 | H334 | Traction detachment of retina |
|  | CIM-10 | H352 | Other proliferative retinopathy |
|  | CIM-10 | H360 | Diabetic retinopathy |
|  | CIM-10 | H36 | Retinal disorders in diseases classified elsewhere |
| **Laser for diabetic retinopathy** | acts | BGNP001 | Chorioretinal photocoagulation of the posterior pole, with monochromatic laser or colored laser |
|  | acts | BGNP003 | Chorioretinal lesion destruction session by laser photocoagulation, using a contact lens |
|  | acts | BGNP004 | Chorioretinal lesion destruction by transpupillary photocoagulation with laser |
|  | acts | BGNP006 | Destruction of chorioretinal lesion by transscleral photocoagulation |
|  | acts | BGNP008 | Chorioretinal photocoagulation of the posterior pole, with argon or diode laser |
| **Diabetes** | Use of the algorithm developed by CNAM^1^ | |  |

CIM10 corresponds to ICD-10 codes

^1^ Caisse Nationale d'Assurance Maladie. Méthodologie médicale de la cartographie des pathologies et des dépenses, version G9 (années 2015 à 2020, Tous Régimes) [Internet]. 2022.Available from: https://assurance-maladie.ameli.fr/sites/default/files/2022_methode-reperage-pathologies_cartographie_0.pdf
