# Supplementary material for: Association of treated neovascular AMD with disability and mood disorders: A longitudinal nationwide study
Source: Acta Ophthalmol. 2026 Apr 18;104(6):e648–54. doi: 10.1111/aos.70130 (PMC13460392; doi:10.1111/aos.70130)
Supplement: Supplementary file 4 [file AOS-104-e648-s001.docx]

Figure S1 – Algorithm used to identify persons with nAMD
